# Higher-order topological insulators in synthetic dimensions

**DOI:** 10.1038/s41377-020-0334-8

**Published:** 2020-07-20

**Authors:** Avik Dutt, Momchil Minkov, Ian A. D. Williamson, Shanhui Fan

**Affiliations:** Ginzton Laboratory and Department of Electrical Engineering, Stanford University, Stanford, CA 94305 USA

**Keywords:** Microresonators, Photonic devices, Other photonics, Nanophotonics and plasmonics

## Abstract

Conventional topological insulators support boundary states with dimension one lower than that of the bulk system that hosts them, and these states are topologically protected due to quantized bulk dipole moments. Recently, higher-order topological insulators have been proposed as a way of realizing topological states with dimensions two or more lower than that of the bulk due to the quantization of bulk quadrupole or octupole moments. However, all these proposals as well as experimental realizations have been restricted to real-space dimensions. Here, we construct photonic higher-order topological insulators (PHOTIs) in synthetic dimensions. We show the emergence of a quadrupole PHOTI supporting topologically protected corner modes in an array of modulated photonic molecules with a synthetic frequency dimension, where each photonic molecule comprises two coupled rings. By changing the phase difference of the modulation between adjacent coupled photonic molecules, we predict a dynamical topological phase transition in the PHOTI. Furthermore, we show that the concept of synthetic dimensions can be exploited to realize even higher-order multipole moments such as a fourth-order hexadecapole (16-pole) insulator supporting 0D corner modes in a 4D hypercubic synthetic lattice that cannot be realized in real-space lattices.

## Opening new dimensions for topological insulators

A theoretical structure that provides extra dimensions to topological insulators could enable novel quantum states to be manipulated. The usual definition of topological insulators is that they act as insulators on the three-dimensional inner bulk, but are highly conductive on their two-dimensional surfaces. However, when photonic topological insulators are constructed, other non-spatial dimensions can be harnessed related to frequency, orbital angular momentum or spin. Shanhui Fan and co-workers at Stanford University, USA, designed photonic higher-dimensional topological insulators made from arrays of ring-shaped resonators. The team showed that each pair of rings acts like a ‘photonic molecule’, generating complex isolated quantum states that could be switched on and off. Their work suggests that these synthetic dimensions could be used to explore exotic new phases of matter, with applications such as quantum computing.

## Introduction

A conventional topological insulator in 2D and 3D supports gapless edge states and surface states, respectively, that are protected against local perturbations by the nontrivial topology of the bulk. The existence of these gapless states, which have dimension one lower than that of the bulk that hosts them, is guaranteed by the bulk-boundary correspondence. Recently, the concept of higher-order topological insulators (HOTIs) has been proposed to generalize this bulk-boundary correspondence, revealing the existence of topological states with dimensions two or more lower than that of the bulk. In general, an *n*th order topological insulator in *D*-dimensions supports (*D-n*)-dimensional topological boundary modes of codimension *n*. The first such prediction was of zero-dimensional zero-energy corner states in a second-order topological insulator with gapped edge states, and the existence of these zero-energy corner states was guaranteed by a quantized bulk quadrupole moment^1^. This theoretical prediction was closely followed by the experimental realization of quadrupole HOTIs in several systems, including bismuth^2^, mechanical metamaterials^3^, acoustics^4,5^, electrical circuits^6^, and photonics^7^. However, both the theoretically proposed and experimentally demonstrated HOTIs have been restricted to real-space dimensions, that is, spatially periodic lattices.

In contrast to real-space dimensions, synthetic dimensions are formed by coupling internal degrees of freedom, which can be, for example, the frequency, arrival time, or orbital angular momentum of photons or the spin of ultracold atoms^8,9^. Introducing coupling between these degrees of freedom then allows the study of higher-dimensional physics in lower-dimensional structures^10–12^. A prime focus of research on synthetic dimensions has been the pursuit of conventional topological phases in simple structures, such as the study of the 2D quantum Hall effect in a 1D real-space array^13–17^ or the study of 3D topological physics in a 2D planar array^18,19^. Additionally, researchers have studied two or more simultaneous synthetic dimensions to implement higher-dimensional physics in essentially 0D systems^20–24^. Since the concept of synthetic dimensions is well suited to the study of topological physics in high-dimensional lattices, a natural question is whether HOTIs can be realized in synthetic space.

Here, we answer this question in the affirmative by constructing a photonic higher-order topological insulator (PHOTI) in synthetic dimensions. Our system consists of pairs of ring resonators that are mutually coupled to form an array, realizing a 1D chain of “photonic molecules”^25,26^. By antisymmetrically modulating the two rings in a photonic molecule at the frequency spacing between the ring modes, we realize a lattice along the synthetic frequency dimension. A 1D array of modulated photonic molecules forms a quadrupole PHOTI in the synthetic frequency dimension, in which we show the excitation of topologically nontrivial corner modes. By changing the phase difference of the modulation between adjacent photonic molecules, we show a phase transition from the topologically protected phase with a nonzero quantized quadrupole moment to a phase with zero quadrupole moment. Additionally, we propose, for the first time, a hexadecapole (16-pole) insulator with topologically nontrivial corner modes by leveraging synthetic dimensions to create a 4D hypercubic lattice that cannot be realized in real-space lattices. Our work illustrates the potential of using the concept of synthetic dimensions to explore exotic new phases, including very-high-order topological insulators in high dimensions.

## Results

### Quadrupole PHOTI

**Fig. 1:**
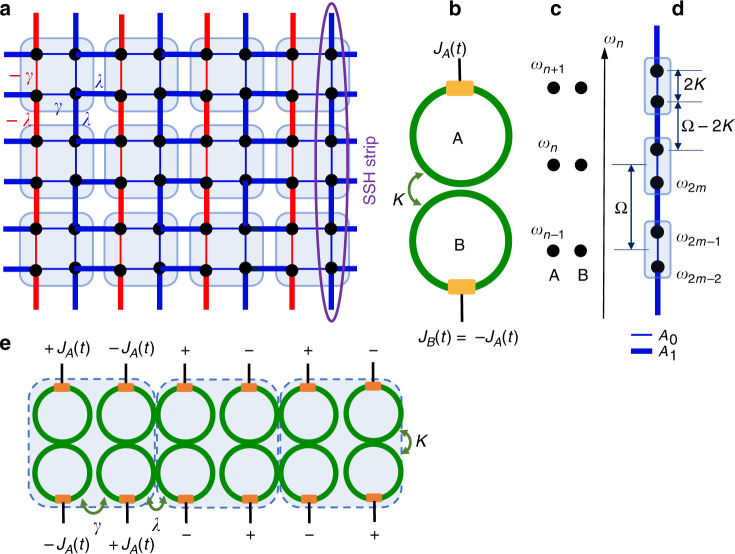
Construction of a quadrupole higher-order topological insulator using a synthetic frequency dimension in a photonic molecule array. **a** Tight-binding lattice of a quadrupole topological insulator. Blue and red lines represent positive and negative real values of the coupling strength. Thin and thick lines represent intra-cell and inter-cell coupling strengths of magnitude *γ* and *λ*, respectively. Each vertical column is an SSH strip with the same coupling strength sign throughout, whereas adjacent vertical lines have a phase offset in the coupling strength of *π*. **b** Implementation of the SSH strip in the synthetic frequency dimension using modulated coupled rings (photonic molecule), with *J*_A_(*t*) as in Eq. (1). **c** Mode structure of the system in **b** with sets of frequency modes separated by the FSR Ω in the absence of modulation and coupling. **d** Mode spectrum of the coupled ring system or photonic molecule. Each set is separated by a frequency difference equal to the coupling constant between the rings, 2*K*. The modulation introduces coupling between the supermodes. **e** Several of the synthetic SSH strips in **b** can be evanescently coupled with alternating coupling strengths *γ* and *λ* to realize the quadrupole HOTI lattice in **a**. We note that the modulation pattern of the unit cell of four rings has a quadrupole nature

Consider the Benalcazar–Bernevig–Hughes (BBH) lattice in Fig. 1a as an example quadrupole higher-order topological insulator (HOTI)^1^. Each vertical 1D strip in this lattice is a Su–Schrieffer–Heeger (SSH) strip^27^, with the alternating values of the couplings *γ* and *λ* representing the intra-cell and inter-cell hopping strengths, respectively. The signs of the couplings along each vertical strip are the same, whereas adjacent lines have a *π* phase difference between the couplings. All the horizontal couplings have positive real values. Thus, there is a magnetic flux *π* through each of the plaquettes.

We show that the model in Fig. 1a can be realized by using the concept of a synthetic dimension. To construct each SSH strip of the quadrupole HOTI, we use a pair of mutually coupled identical ring resonators A and B, each with an electro-optic modulator, as shown in Fig. 1b. Such a pair of photonic cavities, with or without modulation, is often called a “photonic molecule”^25,26,28–31^ in analogy with a diatomic molecule. Each individual ring supports longitudinal cavity resonances at *ω*_*n*_ = *ω*_0_ + *nΩ*, separated by the free spectral range (FSR) *Ω*/2*π* = *v*_g_/*L*, where *v*_g_ is the group velocity of light in the ring, and *L* is the ring length (Fig. 1c). In forming the photonic molecule, the two modes of the two individual rings at the same frequency *ω*_*n*_ hybridize into symmetric and antisymmetric supermodes with frequencies *ω*_*n*−_ = *ω*_*n*_−*K* ≡ *ω*_2*m*_ and *ω*_*n*+_ = *ω*_*n*_ + *K* ≡ *ω*_2*m*+1_, respectively, where *K* is the coupling strength between the rings (Fig. 1d). We neglect dispersion in the ring-to-ring coupling strengths. The frequencies of the photonic molecule in the basis of the symmetric and antisymmetric supermodes thus form a strip with alternating spacings 2*K* and *Ω*−2*K*. By choosing a modulation of the form

1 $$\begin{array}{*{20}{l}} {J_{A}\left( {t,\phi } \right) = 2A_{0}{\cos} \left( {2Kt + \phi } \right) + 2A_{1}{\cos} \left[ {\left( {{\mathrm{\Omega}} - 2K} \right)t+\,\phi } \right];J_{B}\left( {t,\phi } \right) = - J_{A}\left( {t,\phi } \right) } \end{array}$$

one can form a synthetic frequency dimension with alternating coupling strengths *A*_0_ and *A*_1_ (see Supplementary Materials Section I). Note that the coupling strengths achieved in previous work^13,32^ along the synthetic frequency dimension have typically been uniform for a single modulated ring. Here, we use a pair of modulated rings to realize the nonuniform alternating coupling strengths needed for an SSH strip, since the unequally spaced modes can be addressed by the different frequencies of the modulation 2*K* and *Ω*−2*K*. The antisymmetric modulation *J*_*B*_ = −*J*_*A*_ is necessitated by the opposite symmetries of the two supermodes at adjacent frequencies. Throughout most of the paper, we assume

2 $$\begin{array}{*{20}{c}} {A_0,A_1 \ll K < \Omega /4 } \end{array}$$

so that the rotating wave approximation is valid. In Supplementary Materials Section II, we confirm the validity of our synthetic dimension approach in realizing the tight-binding model for the SSH strip when Eq. (2) is satisfied. We also show that the hallmark of the SSH model—the exponentially localized topological edge state—is preserved for moderate modulation strength *A*_1_/*K* = 0.2 even beyond the RWA, although the tight-binding model breaks down. Eventually, the edge state vanishes for ultrastrong modulation *A*_1_/*K* = 1^33,34^.

Next, to form the full 2D lattice of the quadrupole insulator in Fig. 1a, we form a lattice of the pairs of cavities described above, with alternating coupling strengths *γ* and *λ* determined by the respective coupling gaps between the nearest neighbor cavities along the horizontal axis (Fig. 1e). This system is described by a two-dimensional synthetic space with a real space axis (*x*) and a synthetic frequency axis (*m*). The signs of the modulation are switched between adjacent cavity pairs (*ϕ* = 0 and *ϕ* = *π* in Eq. (1)) to implement a flux of *π* per plaquette in the two-dimensional lattice^13,35^. Interestingly, the signs of the modulation in each unit cell of this array of paired resonators follow a quadrupole pattern, as seen in Fig. 1e. The strength of the modulation is chosen to satisfy *A*_0_ = *γ* and *A*_1_ = *λ*, which ensures that the model is isotropic in the magnitude of the coupling strengths along *x* and *m*. Thus, the BBH model is realized, which possesses time-reversal symmetry, two mirror symmetries (along *x* and *m*) and inversion symmetry. Importantly, the two mirror symmetries along the real and synthetic axes do not commute due to the presence of the *π* flux per plaquette, which is ensured by the relative modulation phase between all adjacent pairs of rings along *x* of *π*^1^. This leads to a quantized nonzero bulk quadrupole moment in the system. A deviation of the relative modulation phase from *π* breaks the time-reversal symmetry. Similarly, disorder in the resonance frequencies between adjacent rings breaks the corresponding mirror symmetry. The robustness of higher-order topology to such broken symmetries is studied in the Supplementary Materials Section IV.

### Excitation of corner modes

**Fig. 2:**
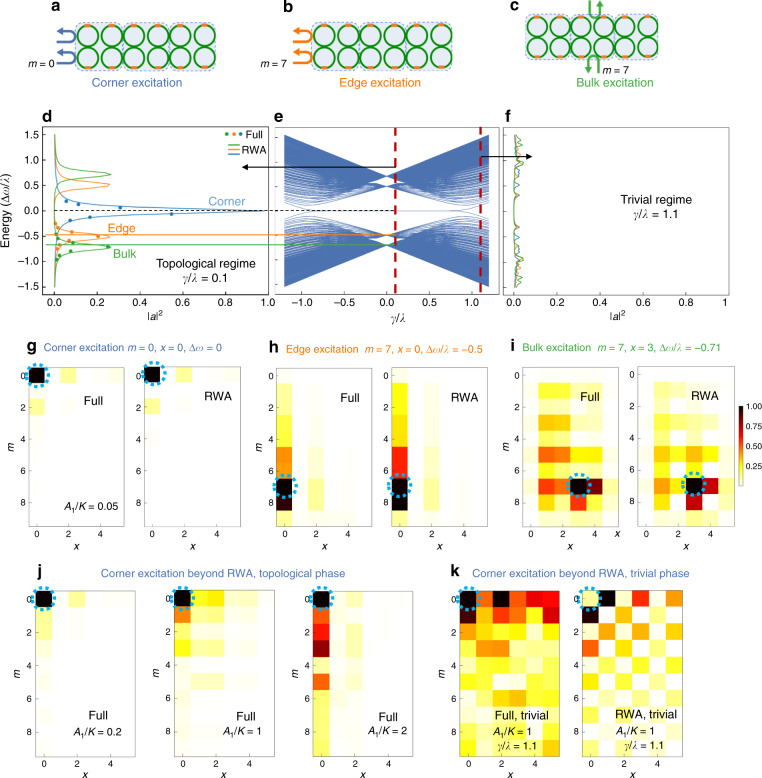
Energy eigenspectrum, edge modes, and corner modes of the synthetic dimension quadrupole HOTI. **a–c** Schematics of selective excitation of corner, edge, and bulk modes in the photonic molecule array using external waveguides. **d** Intensity $\left| a \right|^2$ in the excited rings under various input frequency detunings Δ*ω* for the excitations indicated in **a**–**c**. Peaks appear at input frequency detunings that correspond to the positions of the corner, edge, and bulk modes in **e**. Solid lines represent tight-binding model solutions under the rotating wave approximation [RWA, Eq. (2)], with *γ*/*λ* = 0.1. Dots represent the solutions of the full dynamical coupled-mode equations using the modulation in Eq. (1), with *A*_1_/*K* = 0.05 and *A*_0_/*A*_1_ = *γ*/*λ* = 0.1 **e** Energy eigenspectrum for a large finite lattice of the quadrupole HOTI. For $\left| {\gamma /\lambda } \right| < 1$, the system exhibits topologically protected corner modes pinned to zero energy. For $\left| {\gamma /\lambda } \right| > 1$, the corner modes cease to exist. **f** Same as in **d** but in the topologically trivial regime *γ*/*λ* = 1.1. No peaks are observed in the bandgap, as the corner and edge modes cease to exist. The overall amplitude in the excited rings is lower than that in **c** because the excitation spreads into the bulk. **g–i** Cavity field intensity when exciting the finite lattice at the corner **g**, edge **h** and bulk **i** for *γ*/*λ* = 0.1. The RWA results agree well with the solution of the full dynamical equations. No such corner or edge localized modes were observed in the trivial phase under the same excitations for $\left| {\gamma /\lambda } \right| > 1$. The color scale in the bottom two rows indicates the steady-state field amplitude distribution in the lattice. Blue dashed circles denote the lattice site excited in each case. **j** Lattice field distributions for corner excitation in the topological phase with *γ*/*λ* = 0.1 obtained using the full dynamical equations for increasing values of *A*_1_/*K* = 0.2, 1, and 2, all beyond the validity of the RWA [Eq. (2)]. The field distribution significantly deviates from that for the corner mode excitation based on the RWA (in **g**), but corner localization is still observed for moderate modulation strengths *A*_1_/*K* < 1. **k** Simulations highlighting the difference in the field distributions obtained using the full solution and the RWA upon exciting a corner site in the trivial regime [*A*_0_/*A*_1_ = *γ*/*λ* = 1.1] under ultrastrong modulation [*A*_1_/*K* = 1]

The hallmark of the quadrupole HOTI model described in Fig. 1a is the existence of fourfold degenerate zero-energy corner modes with codimension 2, while the edge modes are gapped, for $\left| {\gamma /\lambda } \right| < 1$. In our implementation with an array of modulated photonic molecules, as shown in Fig. 1e, these midgap corner modes can be excited by coupling external waveguides to the array (Fig. 2a–c). The demonstration of these corner modes then indicates that we have indeed constructed a quadrupole photonic HOTI in synthetic space. Since these corner modes only exist in a finite lattice, we choose *M*_*ω*_ = 10 sites (five pairs of supermodes) along the frequency axis and six rings (Fig. 2a–c) along the real-space horizontal axis for our calculations. Such a termination of the frequency axis can be achieved by strong coupling to a ring with a radius *M*_*ω*_ times smaller than that of the main rings to induce a strong local change in the FSR every *M*_*ω*_ modes^23^. Alternatively, one can engineer the dispersion of the ring waveguide to strongly perturb the FSR beyond the *M*_*ω*_ modes^13^, which makes the modulation in Eq. (1) beyond the finite lattice formed by these *M*_*ω*_ modes off-resonant.

Figure 2d shows the results of exciting the photonic molecule array in the topological phase with *γ*/*λ* = 0.1. We can observe corner, edge, and bulk modes by exciting suitable rings at the appropriate frequency (*ω*_in_ = *ω*_m_ + Δ*ω*), where m denotes the desired frequency mode and Δ*ω* maps to the quasi-energy when this time-modulated system is treated as a Floquet system. Note that selective excitation of a single site in the synthetic lattice requires the excitation of two rings either in phase (+ mode, m even) or out of phase (− mode, m odd), as the modes in each photonic molecule are symmetric and antisymmetric combinations of the isolated ring modes (Fig. 2a–c). For corner mode excitation, we choose the leftmost pair of rings with an excitation frequency *ω*_in_ = *ω*_*m*=0_ + Δ*ω*. We observe a peak for Δ*ω* = 0 because the midgap corner modes are pinned to zero energy, consistent with the eigenspectrum shown in Fig. 2e. The broadening of the peak is due to the coupling to the external waveguides at a rate *κ*_ex_ = 0.03*A*_1_ and due to the intrinsic losses *κ*_in_ = *κ*_ex_ assumed in each ring. In Fig. 2d, dots represent the results of integrating the full dynamical coupled-mode equations using the modulation in Eq. (1), whereas solid lines represent the solutions under the RWA. The two methods agree well with each other since Eq. (2) is satisfied: *A*_1_/*K* = 0.05 ≪ 1 and *K* = 0.15Ω. The corresponding steady-state field amplitude distribution in the synthetic lattice for Δ*ω* = 0 is shown in Fig. 2g, which exhibits strong localization at the corner, with excellent agreement between the RWA and the full solution. For edge mode excitation, we choose the same pair of rings but change *ω*_in_ to *ω*_*m*=7_ + Δ*ω* and observed peaks for Δ*ω*/*λ* ≈ ±0.5. The output amplitude is zero between the peaks, which indicates that the edge modes are gapped. For bulk excitation, we choose a pair of rings in the center of the array and observed peaks at Δ*ω*/*λ* ≈ ±0.71, in accordance with the eigenspectrum in Fig. 2e. The corresponding field amplitude distributions in the synthetic lattice when exciting the edge and bulk modes at their respective detunings Δ*ω* are shown in Fig. 2h, i, with reasonable agreement in all cases between the RWA and the full solution. By contrast, in the trivial regime *γ*/*λ* = 1.1, we do not observe midgap peaks because the corner modes cease to exist—in fact, there are no modes in the bandgap even for a finite lattice (Fig. 2f). Experimentally, the field distributions corresponding to the corner and edge states can be mapped by frequency-resolved detection of the cavity output from the leftmost pair of rings. Heterodyne detection can be used for this purpose, as demonstrated in our recent experiments^22,36^, by beating the cavity output with a frequency-shifted portion of the input laser.

To further probe the validity of the synthetic dimension approach in realizing the quadrupole HOTI, we studied the field distributions under corner excitation for increasing strength of modulation *A*_1_ beyond the validity of the RWA [Eq. (2)], as shown in Fig. 2j, k. For *A*_1_/*K* = 0.2, the steady-state field distribution in Fig. 2j deviates from the RWA solution in Fig. 2g, but corner localization, a signature of nontrivial higher-order topology, is still observable^33^. For ultrastrong modulation [*A*_1_/*K* = 1 and 2], the field significantly penetrates into the edge along the synthetic dimension as the counterrotating terms become non-negligible. The penetration into the bulk along the real-space axis is less significant, as there are no counterrotating terms in that direction that depend on the modulation strength. In Fig. 2k, we show the field distribution in the trivial regime [*γ*/*λ* = 1.1] for ultrastrong modulation. Although the field patterns obtained from the RWA and the full dynamical solution differ, both show significant penetration into the bulk.

### Topological phase transition

**Fig. 3:**
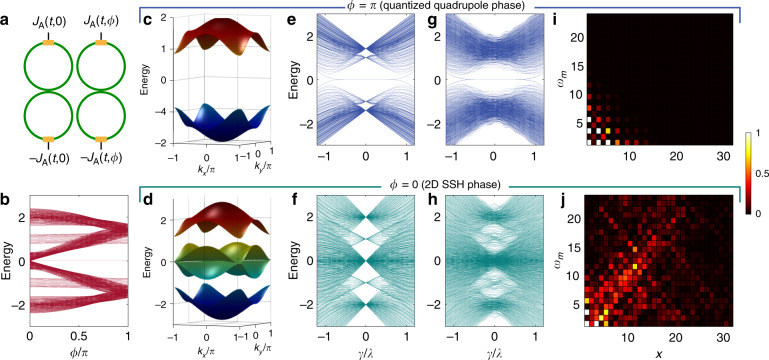
Topological phase transition in the synthetic PHOTI lattice by tuning the phase of the modulation. **a** Unit cell consisting of two photonic molecules with a phase difference between the modulations of *ϕ* [Eq. (1)]. **b** Energy eigenspectrum for a finite lattice with open boundaries in both real dimension *x* and synthetic frequency dimension *m* for various phases *ϕ*, with *γ*/*λ* = 0.20. For *ϕ* = *π*, there is a bandgap that hosts topologically protected corner modes pinned to zero energy. For *ϕ* = 0, no such bandgap exists at zero energy, as confirmed by the bulk band structure in **d**. **c** and **d** Bulk band structures for the quadrupole HOTI (*ϕ* = *π*) and the 2D SSH model (*ϕ* = 0), respectively. All energies are in units of *λ*. In **c**, both bands are doubly degenerate. **e** and **f** Energy eigenspectrum of an ideal finite lattice for *ϕ* = *π* and 0, respectively, without disorder in the couplings. Although corner modes exist in both cases, for *ϕ* = 0, they spectrally overlap with the bulk bands. **g** and **h** Energy eigenspectrum with normally distributed random disorder in the couplings with variance *σ*^2^ = 0.04. Since the lattice with *ϕ* = *π* hosts a quantized bulk quadrupole moment, corner modes are visible in the bandgap in **g**, unlike in **h**. **i** and **j** Steady-state field distribution at the disordered synthetic lattice sites for an excitation with zero detuning Δ*ω* = 0 at the lowest frequency mode *m* = 0 for the leftmost ring, as indicated by the arrows. For the quadrupole PHOTI (*ϕ* = *π*) in the top row, the corner modes are still strongly localized in the presence of disorder. For the 2D SSH phase in the bottom row, disorder in the couplings makes the corner excitation not well localized, with leaking into the bulk. Specifically, the excitation preferentially propagates at ~±45° in the lattice^39^ because the bands in **d** touch at zero energy along the *k*_*x*_ = *k*_*y*_ and *k*_*x*_ = −*k*_*y*_ lines. In **c**–**j**, *γ*/*λ* = 0.4

The concept of a synthetic dimension provides great flexibility in dynamically reconfiguring the hopping amplitudes and phases by changing the strengths and phases of the modulation, respectively. We use this flexibility to show a topological phase transition between the regime with a quantized bulk quadrupole moment and a 2D SSH phase with no quadrupole moment, which occur for *π* and 0 flux per plaquette, respectively. The lattice with zero flux (2D SSH model) possesses all the symmetries of the quadrupole insulator, namely, it is invariant with respect to the translation, reflection (about *x* and *y*) and time-reversal operations. While this ensures that the bulk quadrupole moment is quantized, its value is zero. In fact, there is not even a bulk bandgap at zero energy in this model, meaning that the bulk is not insulating. Our photonic molecule array can be used to implement such a change in flux by changing the relative phase between the modulations on adjacent molecules (Fig. 3a). In Fig. 3, we plot the energy eigenspectrum for various *ϕ*. The bandgap remains open for intermediate values of flux 0 < *ϕ* <*π* but eventually closes for *ϕ* → 0. However, the quadrupole moment is not quantized for intermediate values of flux *ϕ* due to the breaking of the reflection symmetry along the frequency dimension^37^. The bulk band structures for *ϕ* = *π* and *ϕ* = 0 are plotted in Fig. 3c, d. The 2D SSH model with *ϕ* = 0 is not an insulator at zero energy, since the bulk is not gapped for *E* = 0, and although corner modes exist, they spectrally overlap with the bulk excitations. To compare the topological protection of corner modes in the quantized quadrupole phase with *ϕ* = *π* and the 2D SSH phase, we introduce disorder in the couplings. The corresponding eigenspectra (Fig. 3g, h) retain the well-separated midgap corner modes for the quadrupole phase but not for the 2D SSH case. Upon exciting the corner site in the two cases, strong corner localization of the field distribution is observed for *ϕ* = *π* (Fig. 3i), but significant leakage into the bulk is observed for *ϕ* = 0 (Fig. 3j). Leakage into the bulk preferentially occurs along the *k*_*x*_ = *k*_*y*_ and *k*_*x*_ = −*k*_*y*_ directions since the central bulk subbands in Fig. 3d touch at zero energy, which is the energy where the corner modes exist for a finite lattice. This lack of protection of the corner modes for *ϕ* = 0 is expected because the system has zero bulk quadrupole moment and no bandgap at zero energy. Recently, this 2D SSH model without magnetic flux has received some attention^38–40^, because it can be associated with a nonzero 1D Zak phase in both directions. This is in fact what ensures the existence of the corner modes in the system without disorder. However, as we can see in Fig. 3j, these corner states are not as robust as those of the bulk quadrupole insulator phase, which is harder to achieve in real space due to the requirement of negative-valued couplings but is straightforward to achieve using synthetic dimensions.

An alternative way to implement a topological phase transition is to tune the ratio of intra-cell hopping *A*_0_ to inter-cell hopping *A*_1_ in the synthetic frequency dimension by varying the modulation amplitude. This produces an anisotropic 2D SSH model with *π* flux per plaquette, as the hoppings along the real-space axis *x* are fixed. However, the corner modes only exist for *A*_0_/*A*_1_ < 1. As the modulation amplitudes are tuned from *A*_0_ < *A*_1_ to *A*_0_ > *A*_1_, the one-dimensional Zak phase along the frequency dimension becomes topologically trivial, and the corner modes disappear. The Zak phase along the real dimension, however, remains nontrivial, and upon truncation in real space, edge modes still exist at the boundary of the real dimension^38^.

### Octupole and hexadecapole insulators

**Fig. 4:**
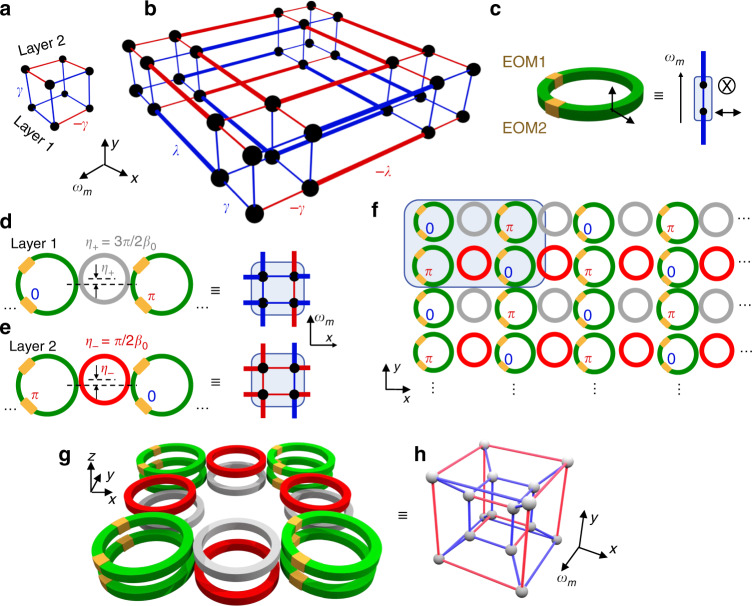
Hierarchical construction of octupole and hexadecapole insulators using synthetic dimensions. **a** Unit cell and **b** tight-binding model of the octupole insulator. Thin lines have coupling strength *γ*, and thick lines have coupling strength *λ*. Blue and red lines represent positive and negative coupling strengths, respectively. **c** Dipole insulator (SSH strip) formed using two polarization modes in a single resonator^25^. EOM1 introduces a frequency offset between the resonances of the two polarizations. EOM2 is modulated by a signal similar to Eq. (1) to form the synthetic frequency dimension spanned by *ω*_m_. **d** Unit cell of the quadrupole insulator formed by coupling two site rings (green) with an auxiliary link ring (gray) with a slightly smaller length and asymmetrically placed with respect to the coupling region with an offset *η*_+_ = *π*/2*β*_0_^41^. This implements layer 1 of the octupole unit cell in **a**. The modulation phases between adjacent site rings differ by *π*. **e** Implementation of layer 2 of the octupole unit cell in **a**. The red link ring implements a negative coupling in real space by having an offset *η*_−_ = 3*π*/2*β*_0_. Negative-valued real-space coupling strengths are needed in our construction of octupole and hexadecapole insulators. **f** 2D lattice of modulated rings with a synthetic frequency dimension forming the 3D octupole insulator in **b**. **g** Array of rings implementing the unit cell of the hexadecapole insulator using two layers of the octupole insulator vertically coupled. The phases of all synthetic and real-space couplings alternate between the two layers. The vertical ring couplings are positive-valued. **h** 4D hypercubic unit cell of the hexadecapole insulator. The inner cube is realized using the bottom layer in **g**, and the outer cube is realized using the top layer

Finally, we show how the concept of synthetic dimensions can be exploited to construct PHOTIs of even higher order, such as an octupole insulator in a 3D cubic lattice and a hexadecapole (16-pole) insulator in a 4D hypercubic lattice supporting corner modes with codimension 4. The unit cell for the octupole insulator cubic lattice is shown in Fig. 4a. It consists of two layers of the unit cell for the quadrupole insulator connected by positive-valued coupling γ^1^. The signs of all couplings are reversed between the two layers (Fig. 4a). The full lattice for the octupole insulator can be created by connecting such unit cells with coupling strength ±*λ* such that a *π* flux is maintained in each plaquette. An example of a small finite lattice for this model is shown in Fig. 4b. Regardless of the multipole order, multipole HOTIs can be viewed as being composed of 1D SSH strips connected in a certain way. In Fig. 4b, for example, a 1D strip in any direction is an SSH chain with either a positive or a negative sign for all of its couplings. The crucial characteristic for each dimension is then whether the couplings of the SSH chains flip sign when the chains are stacked in that dimension (as in the *x*- and *ω*-dimensions in Fig. 4b) or if they are all of the same sign (as in the *y*-dimension). The general rule is that the SSH chains flip sign along all but one of the dimensions.

In the construction of a quadrupole PHOTI, in connection with the experiments in ref. ^22^, we formed an SSH model using two rings and utilized only one of the two polarizations that the ring can support. For the construction of octupole and hexadecapole PHOTIs, since we are using a much larger number of rings, it is of interest to reduce the number of rings used. Therefore, we instead construct the SSH model using only one ring resonator and utilize the polarization degree of freedom. For this purpose, we consider the setup shown in Fig. 4c, where two electro-optic modulators EOM1 and EOM2 are incorporated in a single ring. This setup was previously used in ref. ^25^ for realizing a photonic molecule, but without a synthetic frequency dimension. Here, the two modes forming the SSH unit cell are the polarizations in-plane with the ring and perpendicular to the ring (Fig. 4c). The splitting between the resonance frequencies of these two polarizations is proportional to the voltage applied on EOM1 and can be tuned to 2*K*, similar to Fig. 1d. Next, using EOM2, these polarizations are coupled to each other and to the modes separated by the FSR using the modulation in Eq. (1) with frequency components at 2*K* and *Ω*−2*K*. To facilitate this coupling, the principle axes of EOM2 are at an angle of 45° with respect to those of EOM1.

After realizing the SSH model in a single ring, we form the unit cell of the quadrupole insulator using two such rings, as shown in Fig. 4d. Since layer 2 of the octupole unit cell requires negative-valued couplings in real space, we use off-resonant link rings of a slightly smaller length *L*−*η*, similar to the construction of Hafezi et al. ^41^. The link rings in the second layer are offset from the site rings by *η*_−_ = *π*/2*β*_0_ to realize this negative coupling (Fig. 4e), where *β*_0_ is the propagation constant of the mode at frequency *ω*_0_ in the waveguide forming the ring. We assume *L* ≫ *η* to ensure negligible variation of the coupling phase across the *M*_*ω*_ frequency dimension modes. The signs of the modulation also alternate between each neighboring site ring in the unit cell. Figure 4f shows the entire 2D square array of modulated rings that forms the octupole insulator 3D cubic tight-binding lattice. Thus, the 2D lattice in Fig. 4f with a synthetic frequency dimension forms a quantized octupole insulator supporting midgap corner-localized modes.

Using a similar recipe, we construct a hexadecapole insulator by adding a third spatial dimension to the octupole insulator and switching the signs of all real-space couplings between vertical layers. The unit cell for the hexadecapole insulator has eight site rings with alternating signs of the modulation between adjacent site rings (Fig. 4g). Thus, we form the 4D hypercubic lattice shown in Fig. 4h in real and synthetic dimensions, supporting 0D corner modes with codimension 4, signifying a fourth-order topological insulator. Specifically, the inner cube of the hypercubic lattice in Fig. 4h is formed by the bottom layer of rings in Fig. 4g, and the outer cube is formed by the top layer of rings. The two cubes are connected by positive-valued couplings, as shown in Fig. 4h, which are implemented by vertical coupling between the site rings in Fig. 4g. Such vertically coupled rings have been routinely experimentally realized in silicon photonics and III–V photonics^42–45^. We note that the realization of the hexadecapole insulator is difficult in real space due to the three-dimensional nature of space.

## Discussion

We have introduced the concept of synthetic dimensions for realizing higher-order topological phases supporting quantized bulk quadrupole, octupole, and hexadecapole moments. These phases support topologically protected zero-dimensional corner modes that are robust against disorder in the couplings. We have also shown the excitation of these corner modes in real and synthetic dimensions and a dynamical topological phase transition between a quadrupole insulating phase and a 2D SSH phase. Future work could involve constructing 1D boundary modes of HOTIs, such as chiral hinge states, using similar synthetic-space concepts. Although we focused on a photonic implementation using a synthetic frequency dimension, our approach can be generalized to other degrees of freedom, such as the spin or momentum of ultracold atoms and molecules or the orbital angular momentum of light. Additional frequency dimensions can also be harnessed for this purpose^20,21,24^. Lastly, our proposal is ripe for experimental demonstration using integrated nanophotonic platforms that can modulate resonators at frequencies approaching their FSR, especially in silicon and lithium niobate systems^46–48^.

*Note*: While this manuscript was being prepared, we became aware of a related work using synthetic frequency and orbital angular momentum dimensions^49^.

## Supplementary information

Supplemental material
