# Supplementary material for: Higher-order topological insulators in synthetic dimensions
Source: Light Sci Appl. 2020 Jul 20;9:131. doi: 10.1038/s41377-020-0334-8 (PMC7371732; doi:10.1038/s41377-020-0334-8)
Supplement: Supplementary file 1 — Supplemental material [file 41377_2020_334_MOESM1_ESM.pdf]

Supplementary Material for "Higher-order topological insulators in synthetic dimensions"

Avik Dutt, Momchil Minkov, Ian A. D. Williamson, and Shanhui Fan

Ginzton Laboratory and Department of Electrical Engineering,

Stanford University, Stanford, CA 94305, USA

I. REALIZING AN SSH STRIP USING A MODULATED PHOTONIC MOLECULE

In this section we prove that a modulated photonic molecule can be used to realize an SSH strip along the synthetic frequency dimension. For this purpose, consider the system of a photonic molecule comprising two coupled rings, as in Fig. 2(b). The rings A and B are modulated by signals $J^A(t)$ and $J^B(t)$, respectively.

The coupled amplitude equations for the modal amplitudes α_m and β_m in rings A and B are [1]:

$$\dot{\alpha}_m = i\omega_m\alpha_m + iK\beta_m + i\sum_{m'} J^A_{m'-m}(t)\alpha_{m'} \tag{S1}$$

$$\dot{\beta}_m = i\omega_m\beta_m + iK\alpha_m + i\sum_{m'} J^B_{m'-m}(t)\beta_{m'} \tag{S2}$$

Define c_m and d_m as the modal amplitudes in the symmetric and antisymmetric supermodes,

$$c_m, d_m = \frac{1}{\sqrt{2}}(\alpha_m \pm \beta_m) \tag{S3}$$

Then,

$$\begin{pmatrix}\dot{c}_m\\ \dot{d}_m\end{pmatrix} = i\begin{pmatrix}\omega_m + K & 0\\ 0 & \omega_m - K\end{pmatrix}\begin{pmatrix}c_m\\ d_m\end{pmatrix} + \frac{i}{\sqrt{2}}\sum_{m'}\begin{pmatrix}J^A_{m'-m}(t) & J^B_{m'-m}(t)\\ J^A_{m'-m}(t) & -J^B_{m'-m}(t)\end{pmatrix}\begin{pmatrix}a_{m'}\\ b_{m'}\end{pmatrix} \tag{S4}$$

$$= i\begin{pmatrix}\omega_m + K & 0\\ 0 & \omega_m - K\end{pmatrix}\begin{pmatrix}c_m\\ d_m\end{pmatrix} + i\sum_{m'}\begin{pmatrix}J^S_{m'-m}(t) & J^{AS}_{m'-m}(t)\\ J^{AS}_{m'-m}(t) & J^S_{m'-m}(t)\end{pmatrix}\begin{pmatrix}c_{m'}\\ d_{m'}\end{pmatrix} \tag{S5}$$

where

$$J^{S,AS}_{m'-m}(t) = \frac{J^A_{m'-m}(t) \pm J^B_{m'-m}(t)}{2} \tag{S6}$$

Thus we see that the symmetric part of the modulation $J^S(t)$ couples c_m to $c_{m'}$, and d_m to $d_{m'}$, whereas the antisymmetric part of the modulation $J^{AS}(t)$ couples c_m to $d_{m'}$. If we want to realize an SSH ladder with the two sites being the c and d sublattices, we want to have only c-to-d coupling, both within each cell and between adjacent cells (see Fig. 1(d)). Hence we set

$$J^S_{m'-m}(t) = 0, \quad \text{or} \quad J^A(t) = -J^B(t) \tag{S7}$$

which is the condition imposed in Fig. 1. If supplementary Eq. (S7) is not strictly satisfied, the Hamiltonian will have coupling terms between c_m and $c_{m'}$ and between d_m and $d_{m'}$, which are undesirable in the SSH model. These spurious terms can still be neglected under

the rotating-wave approximation (RWA) if the modulation does not contain frequencies components at the separation between the c_m modes: $m\Omega$, $m \in \mathcal{Z}$. For our work, we satisfy supplementary Eq. (S7) to minimize the use of the RWA for these couplings.

Now, we use $\omega_m = \omega_0 + m\Omega$ and define a gauge transformation,

$$c_m = \tilde{c}_m e^{i(\omega_0 + m\Omega + K)t}; \quad d_m = \tilde{d}_m e^{i(\omega_0 + m\Omega - K)t} \tag{S8}$$

The coupled amplitude equations for $(\tilde{c}_m, \tilde{d}_m)$ take the form, assuming Supplementary Eqs. (5)-(9),

$$\begin{pmatrix} \dot{\tilde{c}}_m \\ \dot{\tilde{d}}_m \end{pmatrix} = i \sum_{m'} \begin{pmatrix} 0 & e^{-2iKt} J^{AS}_{m'-m}(t) \\ e^{2iKt} J^{AS}_{m'-m}(t) & 0 \end{pmatrix} \begin{pmatrix} \tilde{c}_{m'} e^{i(m'-m)\Omega t} \\ \tilde{d}_{m'} e^{i(m'-m)\Omega t} \end{pmatrix} \tag{S9}$$

From Supplementary Eq. (S5) and Fig. 2(d) we see that c_m and d_m differ in resonance frequency by $2K$, and c_m and d_{m+1} differ by $\Omega - 2K$. To realize the SSH model we choose

$$J^{AS}(t) = 2A_0 \cos 2Kt + 2A_1 \cos(\Omega - 2K)t \tag{S10}$$

In the rotating wave approximation (RWA), where we keep only terms with no time dependence in the Hamiltonian of Supplementary Eq. (9), we get

$$\begin{pmatrix} \dot{\tilde{c}}_m \\ \dot{\tilde{d}}_m \end{pmatrix} = i \begin{pmatrix} 0 & A_0 \\ A_0 & 0 \end{pmatrix} \begin{pmatrix} \tilde{c}_m \\ \tilde{d}_m \end{pmatrix} + iA_1 \begin{pmatrix} \tilde{d}_{m+1} \\ \tilde{c}_{m-1} \end{pmatrix} \tag{S11}$$

This realizes an SSH strip with alternating coupling strengths A_0 and A_1, as indicated in Fig. 2(d).

II. VALIDITY OF TIGHT-BINDING MODEL IN SYNTHETIC DIMENSIONS

Next we check the validity of our synthetic dimension approach by comparing the solutions of the tight-binding model derived using the RWA [Supplementary Eq. (S11)] with a real-time simulation of the full dynamics [Supplementary Eq. (S5)] without the RWA. For this purpose we consider a synthetic lattice of 7 frequency modes (14 sites) and assume $J^S(t) = 0$ and $J^{AS}(t)$ as in Supplementary Eq. (S10). Each of the two rings is excited with a frequency $\omega_{\text{in}} = \omega_0 - K$ using an external waveguide with a coupling rate $\kappa_{\text{ex}} = 1/2\tau_p = 4 \times 10^{-3}\Omega$, and we assume the same internal loss rate $\kappa_{\text{in}} = \kappa_{\text{ex}}$ within each ring (critically coupled). We choose $K/\Omega = 0.2$.

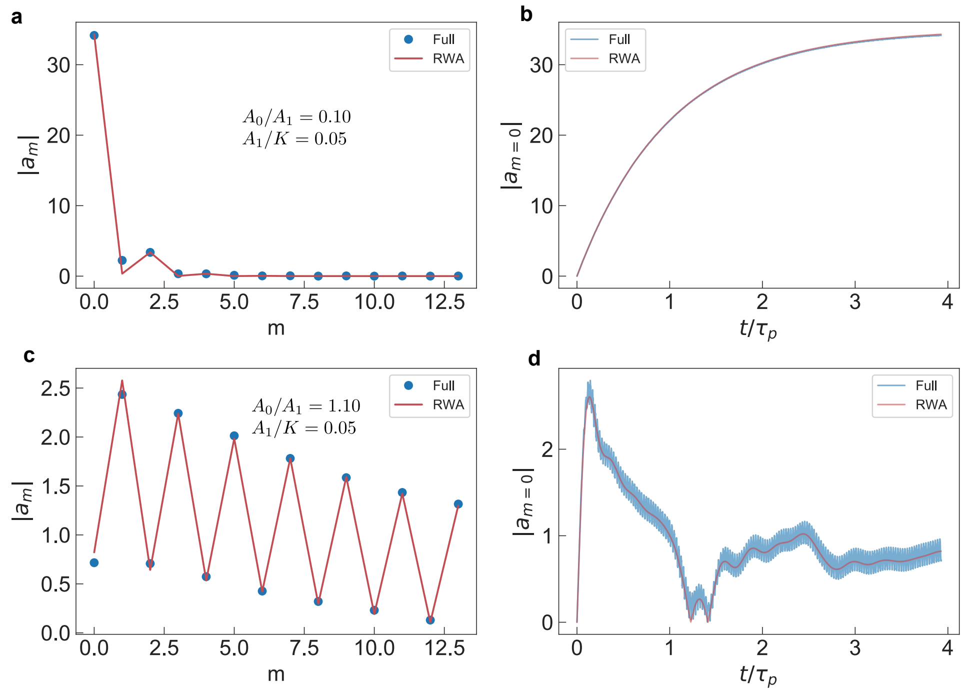

Figure S1. Comparison of the RWA (red) and the full dynamical solution (blue) for the topological regime [$A_0/A_1 = 0.1$ in (a)-(b)]; and the trivial regime [$A_0/A_1 = 1.1$ in (c)-(d)], on exciting the edge of the SSH strip ($m = 0$) in the synthetic frequency lattice. Good agreement is observed between the RWA and the full solution since $A_1/K = 0.05 \ll 1$. The edge mode is excited in the topological regime in (a), which decays exponentially into the bulk. No such exponentially localized edge mode is seen in trivial regime in (c).

In Supplementary Fig. S1(a), (c), we plot the steady-state distribution of $|a_m|$ for $A_1/K = 0.05 \ll 1$, which is well within the validity of the RWA. Here a_m refers to the antisymmetric mode d_m for even m and the symmetric mode c_m for odd m. We observe excellent agreement between the results obtained by full integration of the dynamical equations (shown in blue) and the integration of the RWA equations (shown in red), both in the topological regime [$A_0/A_1 = 0.1$] and the trivial regime [$A_0/A_1 = 1.1$]. The edge mode is excited in (a), which exponentially decays into the bulk. No such localized edge mode is observed in (c). In Supplementary Fig. S1(b), (d) the time evolution of the amplitude of the edge site is plotted. The micromotion within each period of the modulation is visible in (d), but the results of the RWA are nevertheless valid in an average sense both in the trivial and the topological regime. This confirms the validity of our construction of the SSH strip using

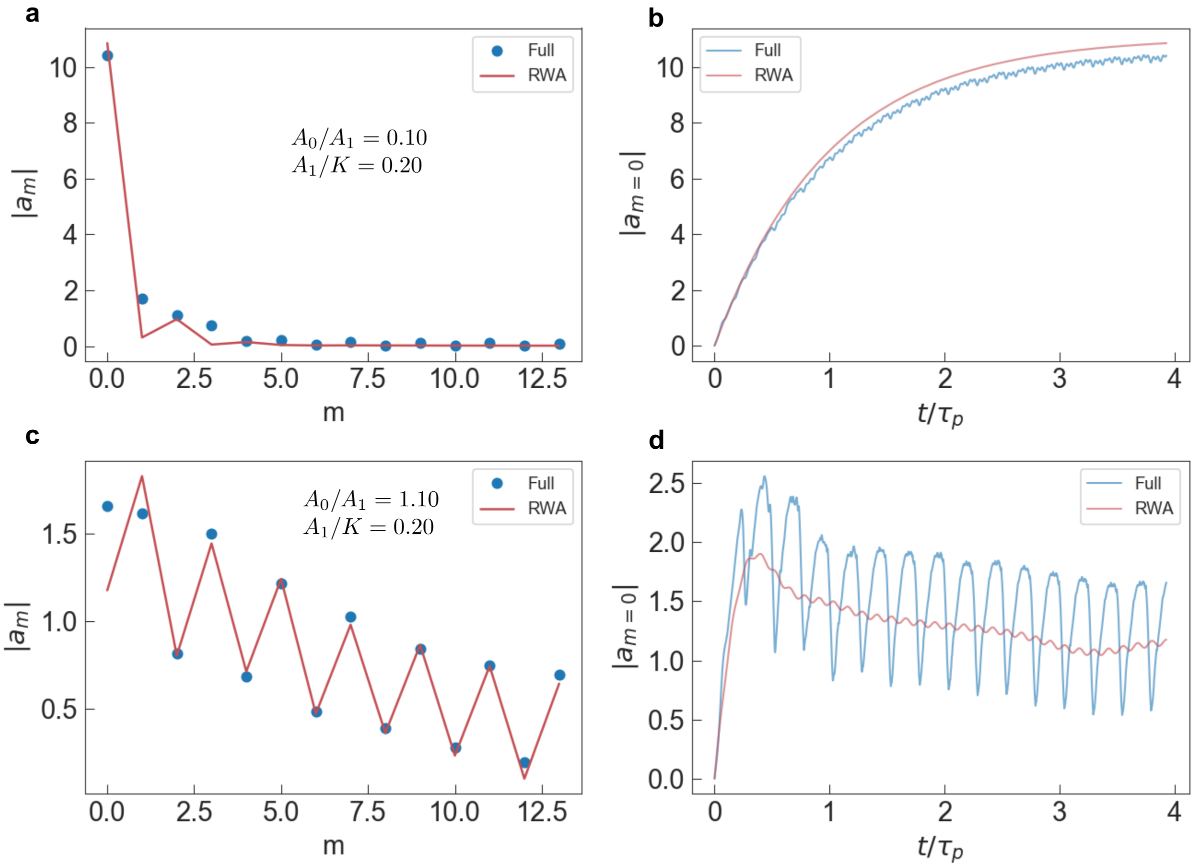

Figure S2. Same as Supplementary Fig.S1 except for a larger $A_1/K = 0.20$. The full solution deviates from the RWA noticably. However, signatures of the edge state are maintained in the topological regime (a), and vanish in the trivial regime (c).

a pair of dynamically modulated photonic molecules, under the condition of Eq. (2) in the main text.

Next, we consider larger values of A_1 in Supplementary Figures S2 $[A_1/K = 0.20]$ and S3 $[A_1/K = 1]$. Although the full solution deviates from the RWA solution for $A_1/K = 0.2$, the exponentially decaying edge state is still observed in Supplementary Figures S2(a) (topological regime), and not observed in the trivial regime [panel (c)]. On the other hand, for even stronger modulation amplitude $A_1/K = 1$ shown in Supplementary Fig. S3, the exponentially localized edge state is not observed even for the expected topological regime $[A_0/A_1 = 0.1$, panel (a)]. These conclusions are in agreement with previous work in real-space topologically nontrivial lattices undergoing ultrastrong modulation [2].

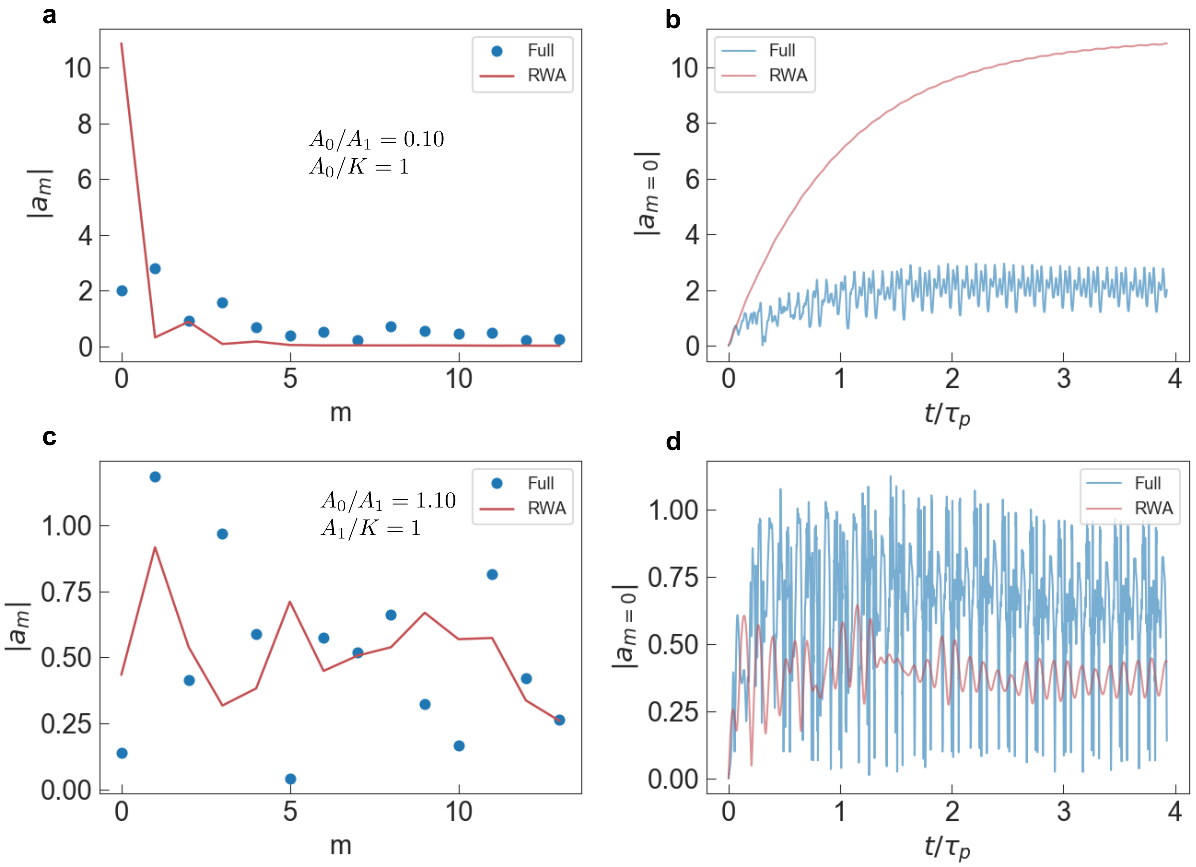

Figure S3. Same as Supplementary Fig.S1 except for a very strong modulation amplitude $A_1/K = 1$. The full solution is starkly different from the RWA solution. The full solution does not show exponential localization of the edge state even for the topological regime (a).

III. BOUNDARY ALONG THE SYNTHETIC FREQUENCY DIMENSION

The existence of a boundary along both the real and synthetic dimension is essential to the identification of corner states in a quadrupole HOTI, as described in the main text. While the boundary along the real horizontal dimension is naturally formed by choosing a finite number of rings, the frequency dimension boundary is less straightforward to construct. Here we provide a schematic of how such a boundary can be constructed. Consider a main ring as shown in Supplementary Fig. S4 with a circumference length L_0, with an effective index n_0. Neglecting group-velocity dispersion, the ring supports longitudinal frequency modes at frequencies $\omega_m = m\Omega_R$, where $\Omega_R = 2\pi c/n_0 L$ is the free-spectral range (FSR). By strongly coupling an auxiliary ring made of the same material as the main ring, with

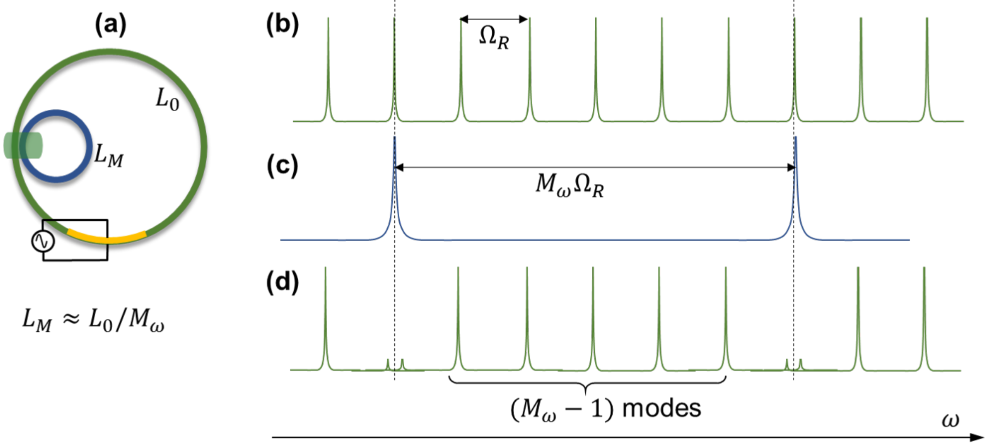

Figure S4. Schematic of constructing a boundary along the synthetic frequency dimension to realize a finite lattice in a single ring. (a) An auxiliary ring (blue) with a length L_M is coupled strongly to the main ring of length L_0. (b) Frequency modes of the main ring. (c) Frequency modes of the auxiliary ring. (d) Frequency modes of the coupled ring system, showing mode-splitting and hybridization at the artificial boundaries. A finite $(M_\omega - 1)$-site lattice is formed.

a length $L_M = L_0/M_\omega$, whose modes are separated by an FSR $\Omega'_R = M_\omega \Omega_R$, one can selectively perturb every M_ω^{th} mode of the main ring. Both the position and the extinction of these perturbed resonances are modified, and hence the modulation is unable to couple beyond the boundary formed by the $M_\omega - 1$ modes. Thus, a finite number of sites along the synthetic frequency dimension is realized.

IV. EFFECT OF LOSSES AND IMPERFECTIONS

In this section, we discuss the influence of losses, and imperfections in resonance frequencies and modulation frequencies on the response of the quadrupole HOTI. Scattering and absorption losses broaden the linewidth of the excited corner and edge states (see Fig. 2d). If the loss rate is significantly lower than the other energy scales in the system such as modulation strength and the inter-resonator couplings ($\kappa_{\text{in}} \ll A_0, A_1, \gamma, \lambda, K, \Omega$), the higher-order topological effects we predict are retained.

Fig. S5 shows the response of the quadrupole HOTI to variations in the resonance frequencies of the rings arising from e.g. fabrication imperfections. We assume a randomly distributed variation in resonance frequencies between adjacent horizontal rings in the range

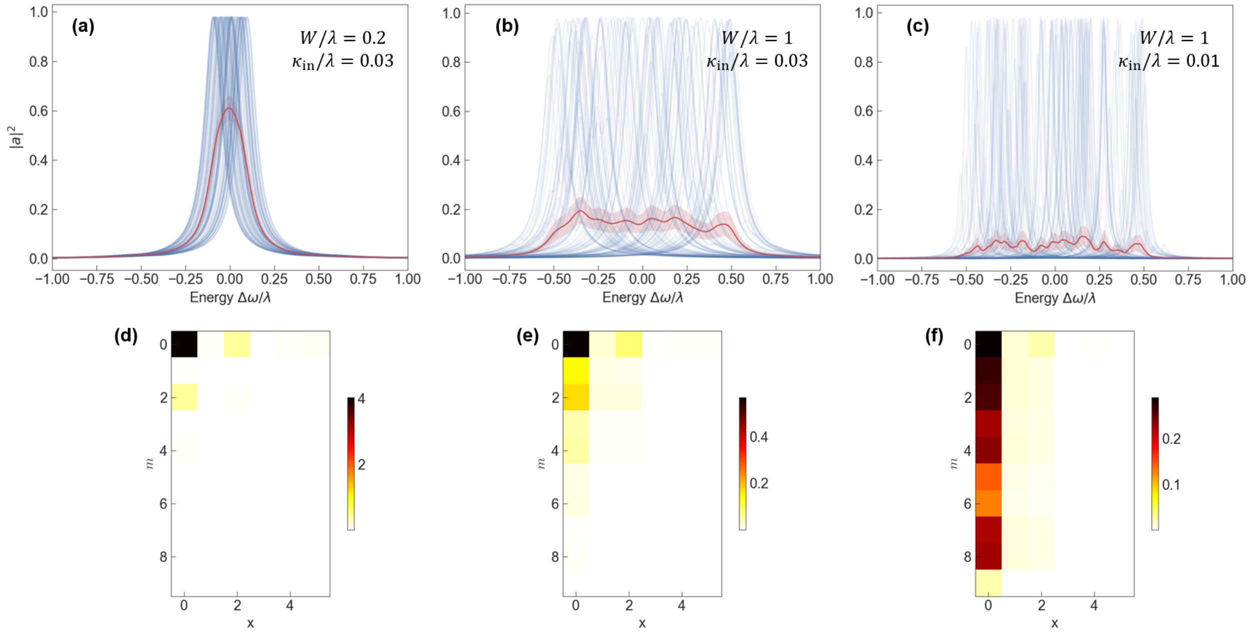

Figure S5. Effect of relative shifts in resonance frequencies on the excitation of corner modes for various values of disorder strength W and loss rate κ_{in}. The variation of resonance frequencies between rings is randomly sampled from a uniform distribution with $\delta\omega_x/\lambda \in [-W/2, W/2]$. Blue lines in (a)-(c) denote intensities in the excited corner site for 100 individual realizations of disorder. Red shaded lines represent the mean response of 100 realizations with 95% confidence interval. Bottom panels depict field amplitude distributions for a representative sample from the corresponding top panel. While the corner mode peaks are not pinned to zero energy in (a)-(c), the exponentially localized corner mode is still excited well for low disorder strengths as in (d). Increasing the disorder strength W [in (b)] causes excitation of an edge-like mode in (e), as edge mode frequencies are shifted towards zero energy. This effect becomes even more pronounced on reducing the loss rate κ_{in}, as in (c) and (f). Note the different colorbar scales between (d)-(f), showing a lower amplitude of excitation on increasing disorder and reducing loss rates.

$\delta\omega_x/\lambda \in [-W/2, W/2]$ for $W = 0.2$ (panel (a), (d)) and 1 (panel b,c,e,f). The position of the peak corresponding to the corner mode varies significantly with this disorder between the rings, since the disorder breaks the mirror symmetry of the lattice and the corner mode is no longer pinned to zero frequency ($\Delta\omega = 0$). Nevertheless, corner state excitation is still robust if the disorder-induced frequency shift is within the linewidth. Hence, for increasing disorder strengths W and for decreasing linewidth κ_{in}, the corner state ceases to be excited,

and the amplitude of the coupled light in the corner site decreases. The field distributions observed in Supplementary Fig. S5(e) and (f) can be understood by noting that, in the presence of increasing disorder between resonance frequencies of adjacent rings, light propagation is suppressed along the real axis x. However, light can continue to propagate along the synthetic frequency dimension because the modulation-induced coupling is still uniform in each ring. We emphasize that this on-site disorder is significantly different from disorder in the couplings (Fig. 3 in the main text), in which case the corner modes are pinned to zero frequency ($\Delta\omega = 0$, Fig. 3g) even for very strong disorder strengths.

We verified in simulations that relative shifts of resonant frequencies have a similar effect on the zero-flux 2D SSH phase as the π-flux quadrupole phase. Thus the relative shifts do not affect the topological phase transition. The major protection offered by the quadrupole phase is with respect to disorder in the coupling strengths, as shown in Fig. 3 of the main text.

If the modulation frequencies are not exactly matched to $2K$ and $\Omega - 2K$, an effective electric field (that is, a linearly tilted on-site potential) is formed along the synthetic frequency dimension, which breaks the translational symmetry of the frequency axis. This aspect has been previously studied by Yuan and Fan in the context of Bloch oscillations and frequency translation [3]. Provided the deviation from the ideal modulation frequencies is sufficiently smaller than the linewidth of each ring resonance ($= \kappa_{\text{in}} + \kappa_{\text{ex}}$), the signatures of quadrupole higher-order topology are retained in the system.

SUPPLEMENTARY REFERENCES

[1] Dutt, A. *et al.* Experimental band structure spectroscopy along a synthetic dimension. *Nat. Commun.* **10**, 3122 (2019).

[2] Yuan, L. & Fan, S. Topologically nontrivial Floquet band structure in a system undergoing photonic transitions in the ultrastrong-coupling regime. *Phys. Rev. A* **92**, 053822 (2015).

[3] Yuan, L. & Fan, S. Bloch oscillation and unidirectional translation of frequency in a dynamically modulated ring resonator. *Optica* **3**, 1014–1018 (2016).
